# Commentary: At least eighty percent of brain grey matter is modifiable by physical activity: a review study

**DOI:** 10.3389/fnhum.2018.00195

**Published:** 2018-05-08

**Authors:** Irene Esteban-Cornejo, Andrés Catena, Charles H. Hillman, Arthur F. Kramer, Kirk I. Erickson, Francisco B. Ortega

**Affiliations:** ^1^Center for Cognitve and Brain Health, Department of Psychology, Northeastern Unviersity, Boston, MA, United States; ^2^PROmoting FITness and Health through Physical Activity Research Group, Department of Physical Education and Sport, Faculty of Sport Sciences, University of Granada, Granada, Spain; ^3^Department of Experimental Psychology, Mind, Brain and Behavior Research Center (CIMCYC), University of Granada, Granada, Spain; ^4^Department of Physical Therapy, Movement & Rehabilitation Sciences, Northeastern Unviersity, Boston, MA, United States; ^5^Beckman Institute, University of Illinois at Urbana-Champaign, Champaign, IL, United States; ^6^Brain Aging & Cognitive Health Lab, Department of Psychology, University of Pittsburgh, Pittsburgh, PA, United States; ^7^Department of Biosciences and Nutrition, Karolinska Institutet, Huddinge, Sweden

**Keywords:** exercise, physical activity, brain, hippocampus, lifespan

The brain is one of the most complex and poorly understood organs of the human body. Understanding the function and complexity of the brain is considered as one of the main challenges for the twenty-first century. Almost a decade ago, in a landmark review, Hillman et al. concluded that there was converging evidence at the molecular, cellular, systems, and behavioral levels that physical activity is beneficial to the brain, however there were many important questions that remained unanswered (Hillman et al., 2008). Since that time, a growing number of publications have focused on this topic, requiring comprehensive reviews that help the field to better understand what is known and what remains to be known on the relationship of physical activity to the brain.

In a recent review published in *Behavioral Brain Research* (Batouli and Saba, 2017), Batouli and Saba concluded that “*at least 80% of brain gray matter is modifiable by physical activity*.” While we believe that this review was well-conducted and well-intentioned, we disagree with the overall conclusion statement because as it overstates the extant findings. The authors summarized the brain areas and the total number of times that each area was reported in individual studies, and then created a single map containing these brain regions. It was the extensive map of regions that the authors used to reach their conclusion. However, such an approach may be greatly misleading. That is, the authors did not emphasize or take into account whether associations for a particular brain region were consistent across studies. In fact, only ~10% of all reported regions were consistently related to physical activity across the reported studies. Specifically, in reference to their table there were only a handful of regions that were consistently reported across numerous studies including the hippocampus (14 times), three frontal cortical regions (i.e., superior frontal gyrus, 9 times; dorsal premotor cortex, 6 times; supplementary motor area, 5 times), two parietal regions (i.e., precuneus, 7 times; inferior parietal gyrus, 5 times) and one temporal region (middle temporal gyrus, 9 times) (Batouli and Saba, 2017). In formulating their conclusion, the authors simply aggregated all reported brain regions to create a single map, ignoring the heterogeneity of findings and then concluding a suspiciously high percentage of 80% of gray matter as modifiable by physical activity. In fact, the map provided in Figure 2 overlooks not only the number of times that a region was reported but also *t*-values, cluster (*K*) sizes, specific coordinate locations and/or correction for multiple comparisons. For example, if a region was reported once with a *K* ≈ 40, *t* ≈ 1.5 and an uncorrected *P-*value, it was given the same weight in their map as a region that was reported several times with a *K* ≈ 500, *t* ≈ 5.5 and a corrected *P-*value. Accordingly, the authors were not rigorous in their analyses, which led to an overstatement of the extant findings in the literature.

Two additional concerns warrant consideration. First, the characterization of the physical activity measure/outcome used in the review was incorrect, as physical activity and physical fitness, which are different constructs, was confused. Physical activity is a behavior describing any bodily movement produced by skeletal muscles that requires energy expenditure, while physical fitness is a health-related marker describing a set of attributes (i.e., cardiorespiratory fitness, muscular strength, and speed-agility) that people must achieve, which enables them to complete activities of daily living (Caspersen et al., 1985). Second, the brain morphology measures were also convoluted across the studies as they included both cortical volume and cortical thickness. By definition, cortical thickness is the distance between white matter and pial surfaces and cortical volume is a product of thickness and surface area. As such, the different brain outcomes must be considered as separate morphometric measurements. In fact, findings suggest that higher cortical volume but lower cortical thickness is related to better intelligence and these associations may fluctuate throughout the lifespan (Schnack et al., 2015).

Collectively, the issues we raise point to serious inaccuracies and shortcomings that must be considered to more adequately interpret the claims made by Batouli and Saba (2017). In our view, a more appropriate conclusion would be: “several studies demonstrated that specific brain regions are susceptible to the influence of physical activity as denoted by changes in cortical volume and cortical thickness across the lifespan and within different populations. As such, it is difficult to formulate a conclusive statement regarding the total percentage of the brain that can be modified by physical activity. More studies are warranted to shed further light on this topic, specifically, in younger populations in which a markedly smaller proportion of studies exist.”

## Author contributions

IE-C, AC, and FO Contributed conception and design of the study. IE-C wrote the first draft of the manuscript. IE-C, AC, FO, CH, KE, and AK Wrote sections of the manuscript. All authors contributed to manuscript revision, read and approved the submitted version.

### Conflict of interest statement

The authors declare that the research was conducted in the absence of any commercial or financial relationships that could be construed as a potential conflict of interest.
